# Posttranscriptional Gene Regulation: Novel Pathways for Glucocorticoids’ Anti-inflammatory Action

**Published:** 2012-04-30

**Authors:** Cristiana Stellato

**Affiliations:** Department of Medicine, University of Salerno, Italy

**Keywords:** Inflammation, Glucocorticoids, RNA-binding Proteins, microRNA

## Abstract

Posttranscriptional gene regulation (PTR) is a fundamental biological process that integrates with the master transcriptional control of gene expression, in ways that only in the last decade have been increasingly understood [1, 2]. While epigenetic and transcriptional events shape cell response qualitatively, deciding the pattern of gene expression to ‘switch on or off’ in response to endogenous or environmental triggers, the key task of PTR is to act as a ‘rheostat’ and rapidly adapt the cellular response by providing the appropriate amplitude and timing to the protein expression patterns [3, 4]. The pivotal role of this mechanism comes to the forefront in inflammatory and immune response, where the changes in amplitude and duration in the expression of dangerous and protective genes are in delicate balance, and are critical in determining either the successful resolution of the immune response or its chronic overexpression [5]. This brief review introduces members of the main classes of molecules mediating the cytoplasmic arm of gene regulation, namely RNA-binding proteins and micro-RNA (miRNA), and summarizes experimental data that underscore the role of these molecules in the pathophysiology of chronic inflammation, as well as their promising value as mechanisms conveying the anti-inflammatory effect of synthetic glucocorticoids.

## I. INTRODUCTION

In the last decade, seminal studies have identified RNA-binding proteins (RBP) and microRNA (miRNA) as key mediators of PTR [6–8]. Both classes of molecules are regulated by the same signaling pathways – chiefly MAP kinases - that modulate transcriptional regulation, underscoring the highly integrative nature of the nuclear and cytoplasmic gene regulatory events[9, 10]. They are among the most abundant and well-conserved eukariotic genes and share the property to coordinately regulate multiple genes through binding to conserved nucleic acid sequences. Often co-localized in cytoplasmic foci of mRNA metabolism (P bodies and stress granules), they can regulate each other’s effects by competing for binding or complementing the binding of one another [11, 12]. As studies are increasingly revealing the complex interactions between these determinants of posttranscriptional gene regulation and their gene targets, the understanding of their potential involvement in the pathophysiology of many human chronic inflammatory diseases is a key task for the generation of novel therapeutic interventions.

### 1. Molecular Mediators of Posttranscriptional Gene Regulation in Inflammation: RNA-binding proteins and micro-RNAs

Since their generation in the nucleus, mRNA molecules are kept as components of ribonucleoprotein (RNP) complexes, in association with a host of RNA-binding proteins (RBPs) that dynamically interact with the mRNAs and modulate their fate: from their correct splicing and nucleocytoplasmic transfer, to their turnover and translation rates [13]. Several conserved regions within the mRNA sequences, where RBPs associate with specific RNA Recognition Motifs (RRMs), have been identified and recently defined as USER (Untranslated Sequence Elements for Regulations), as they are preferentially –though not exclusively - located in the mRNA untranslated regions (UTR), particularly in the 3’UTR [1, 2, 14]. Among these, the Adenylate-Uridylate-Rich Elements (ARE) are the most conserved and well-characterized regulatory elements mediating changes in mRNA turnover and translation in immune and inflammatory genes [15]. ARE-mediated gene regulation has been described for inflammatory genes such as TNF-α, *IL2, IL3, IL-6, GM-CSF,COX-2, INOS, IL4,* and *IL13* and many more including numerous chemokines*,* although other USER have been found to be important, especially for T cell-derived genes [14]. Genome-wide studies examining the transcript pools selectively associated with distinct RBPs have established that functionally related mRNAs that share a USER, such as the ARE, can be coordinately regulated by one or more cognate RBPs [1, 16]. In order to regulate their targets’ mRNA stability and/or translation, multiple ARE-binding proteins bind dynamically to the mRNA targets following the cues of signaling pathways triggered by proinflammatory stimuli [13] and acting either in a cooperative or exclusive fashion.

Exemplary of these complementary actions are the two ARE-binding proteins HuR and tristetraprolin (TTP). They both regulate mRNA transcripts binding to a group of heterogeneous and distinct, but partially overlapping ARE highly conserved in the 3’UTR of inflammatory and immune genes’ mRNAs, such as TNFα, IL-6, GM-CSF, COX-2, INOS, CCL2 and more [17–19].

While the ubiquitous RBP HuR is functionally characterized mainly as a positive regulator of mRNA stability and translation, TTP limits the inflammatory response by accelerating the mRNA decay of its targets [13]. In particular, cell activation induces HuR nucleocytoplasmic shuttling [20–24], an event reflecting its functional activation, which leads to increased protein output for several inflammatory genes, by making their mRNA more stable and/or by increasing their translation rates [22, 25–31] acting through multiple, often independent mechanisms yet to be fully characterized [32].

An important functional counterpart of HuR-mediated increase in inflammatory gene expression is TTP. Tristetraprolin is a member of a small family of CCCH zinc finger proteins that include TTP, Butyrate-response factor (BRF)-1 and BRF-2 [33]. Tristetraprolin promotes mRNA decay through binding of its zinc finger RRM domain to an ARE consisting in adjacent UUAU/UUAU half-sites [34–36]. Stress and inflammatory stimuli induce TTP as an immediate early response gene in numerous cell types, including T cells, macrophages, epithelial cells and fibroblasts, where it predominantly resides in the cytoplasm [37]. TTP is a potent endogenous negative regulator of TNFα-induced inflammation through a negative feedback loop on its activity: in fact, TNF-α induces TTP synthesis, which in 2. turn leads to rapid destabilization and decay of TNF-α mRNA [38]. Besides TNF-α, TTP also increases the 3. mRNA decay of many targets regulated in opposite 4. fashion by HuR [17, 39–42]. Additional TTP-regulated transcripts have been identified in a genome-wide study of mouse embryonic fibroblasts (MEFs) isolated from TTP-knockout (TTP^−/^) mice [18], and in mouse macrophages in which TTP expression was silenced [43]. The importance of TTP in limiting the inflammatory response has been clearly demonstrated also *in vivo* using TTP-deficient mice, which develop severe inflammatory arthritis, autoimmune dysfunction and myeloid hyperplasia through the deregulated expression of TNF-α and GM-CSF [44].

Micro-RNAs (miRNA) are small (approximately 20–25 nucleotides long), endogenous RNAs encoded by one of the largest and most conserved family of genes in eukariotes, which regulate gene expression at a post-transcriptional level mainly with a repressive outcome [45], though miRNA-mediated increase in gene translation has also been reported in cell-cycle processes [46]. It is estimated that up to one third of the human genome may be subject to regulation by miRNAs [47]. Such large control is exerted through the coordinate effect of a single miRNA on multiple transcripts, which are often functionally related, which harbor the seed regions and display other complex structural features dictating functional miRNA:mRNA interaction. Furthermore, a single transcript is targeted by multiple miRNAs, such that a highly combinatorial miRNA profile is what ultimately fine-tunes the protein levels of a high number of targets. MiRNAs act through multiple mecanisms, mediating either mRNA degradation or translation repression of their targets, though they also convey indirect effects on epigenetic and transcriptional regulatory factors [48, 49]. Research in the past decade has revealed their key role in fundamental biological processes such as cell development, proliferation and apoptosis [50], and consequently the immune system and the inflammatory response has resulted to be greatly influenced by miRNA functions[51–56]. Alteration of miRNA expression/function has been increasingly revealed in disease pathophysiology, including inflammatory and allergic diseases [55, 57–60]. As such, modulation of miRNA function, either with miRNA mimics or antagonists (denominated antagomiRs [53]), has increasingly pursued as a strategy to convey therapeutic effect [61, 62]. Importantly, recent studies show that miRNA and RBP can interact through common or overlapping site(s) of their target mRNAs, and their interaction can bring either cooperative or antagonistic outcomes [11, 63, 64]. Thus, it is critical to understand not only how these regulatory factors are modulated by immune activation, but also how their relative contribution is influenced by their relation with other components of the ribonucleoprotein complex.

### 2. Does deregulation of PTR impair the resolution of the inflammatory response?

Despite great advancements in the understanding of the basic mechanisms conveying PTR in inflammation [3], evidence of the pathophysiological impact of PTR in human allergic, infectious and inflammatory diseases is lagging behind - although data pointing at its importance are accumulating. To begin with, pro-inflammatory signaling pathways known to be activated in allergic and inflammatory diseases, such as the stress-activated MAP kinases, are now known to be key regulators of PTR and in particular, of the activation and function of RBPs [13, 65]. For example, in asthmatic patients, the ERK1/2 and p38 pathways, which regulate key cell secretory function and proliferation, are strongly upregulated in airway epithelium and BAL macrophages, and their increase correlates with asthma severity and response to GC therapy [66, 67]. These two pathways are established, potent regulators of TTP and HuR, by inhibiting the first and activating the latter [68, 69]. These evidences strongly suggest that alteration of the components within the ribonucleoprotein complex that regulates mRNA decay and translation may play a pathogenetic role in chronic inflammatory disorders such as asthma or rheumatoid arthritis. It can be hypothesized that inflammatory and/or microbial triggers may produce an aberrant shift of the PTR balance towards increased mRNA stability and translation of the gene pool coordinately regulated through these mechanisms, determining an increase in the amplitude and prolongation in time of the gene expression profile defined transcriptionally.

### 3. Posttranscriptional mechanisms in glucocorticoid’s anti-inflammatory action: a novel focus for anti-inflammatory strategies

It is firmly established that the mechanism of the antiinflammatory action of synthetic glucocorticoids (GC) critically relies on their ability to influence gene expression through transcriptional regulation [70]. More recent data, however, indicate that control of posttranscriptional pathways could constitute a critical component of an efficient anti-inflammatory action [71, 72]. Understanding the pathophysiological role of PTR mediators, such as RBPs and miRNAs, and to what extent they may convey an anti-inflammatory function may lead to novel anti-inflammatory strategies.

GC have been known to accelerate the mRNA decay of a growing number of cytokines, chemokines and other pro-inflammatory molecules, but the molecular mechanisms by which GCs act on post-transcriptional events are still very poorly understood [71]. Glucocorticoids can impact PTR in different ways. An important indirect effect occurs through the induction by GC of MAP Kinase Phosphatase-1 (MKP-1), a phosphatase that inhibits p38 MAP kinase activity, which induces stabilization of many mRNA species relevant to inflammation (cytokines, chemokines, cyclooxygenase-2) [73]. Mouse lacking MKP-1 demonstrate a diminished response to GC [74].

Studies conducted by our laboratory and others indicate that GC induce TTP [75][19]. In our study, genome-wide analysis using mouse embryonic fibroblasts (MEF) from TTP^−/−^ and wild type (WT) littermates revealed that GC-mediated changes in gene expression were strikingly dependent from TTP, with a loss of GC sensitivity for more than 80% of genes that were GC-responsive in WT MEFs [19]. We confirmed the loss of GC-mediated action for several TTP targets in cultured human primary bronchial epithelial cells, using siRNA-mediated TTP ablation. Conversely, in a mouse model of LPS-induced peridontal inflammation, TTP overexpression displayed an antiinflammatory effect through accelerated turnover of TNF-α mRNA [76]. These studies indicate that the impact of posttranscriptional regulation in the mechanism of anti-inflammatory action of GCs is larger than ever appreciated and it involves molecules, like TTP, that directly modulate mRNA stability. However, As TTP is regulated by TNFα and other inflammatory stimuli both at the level of expression *and* functional state by MAP kinases, which functionally inactivate TTP [69, 77–79], in order to exploit TTP-mediated action GC would need to increase TTP levels and regulate its functional state, by controlling the inflammatory signaling that suppress it or by supporting the cellular pathways that counteract the inflammation. Therefore, we are currently testing the hypothesis that if TTP would be increased in level *and* functionally active under GC control, its physiologic role in the termination of the inflammatory response would contribute significantly to the GC-mediated shutdown of inflammatory cytokines, by allowing increased mRNA decay for the many ARE-bearing inflammatory transcripts under TTP control. More recently, we and others [80, 81] have characterized a novel cytoplasmic role of the glucocorticoid receptor (GR) in posttranscriptional gene control, describing that in airway epithelial cells, GR may also function as an RNA-binding protein. Proteins functionally characterized as transcription factors, such as the GR, and RBPs have several features in common, such as stimulus- or ligand-induced nucleocytoplasmic shuttling function and the ability to coordinately regulate multiple genes through binding to conserved nucleic acid sequences. Some regulatory factors can bind to both DNA and RNA, thus influencing both transcription and mRNA fate. For instance, the zinc finger protein NF-90 can regulate transcription of IL-2, but can also bind to and stabilize the IL-2 mRNA [82]. Initial studies done in rat smooth muscle cells reported that the GR interact with the mRNA of the chemokine CCL2, and that the presence of the GR in the RNP complex was necessary for its degradation to occur [80]. work identified a novel role of GR in mRNA turnover, and raised questions about the scope and mechanisms underlying this process. Given the importance of GC action in modulating inflammatory gene expression in human airway epithelium, and given the relevance of other RBPs, such as TTP, in GC action [19], we sought to investigate whether the GR would act as an RBP in human airway epithelial cells, reasoning that, as RBPs can coordinately regulate subsets of functionally related transcripts that share a common recognition motif, such action may regulate not only few targets but a substantial subset of genes[1]. We first identified the association of GR with the chemokines’ CCL2 and CCL7 mRNAs by immunoprecipitation of ribonucleoprotein complex (RNP-IP) and biotin pull-down in the human airway epithelial cell line BEAS-2B. In the case of CCL2, the binding site was mapped to the 5’UTR of the transcript. Furthermore, we aimed at identifying the full complement of transcripts that could bind to GR using a ribonomic approach, and computationally searched for a common GR recognition motif within the identified GR target transcripts. We demonstrate that the GR in human lung epithelial cells is capable of associating with almost 500 transcripts, and we validated such association for a subset of them. Finally, we identified by computational analysis a novel, GC-rich motif, for which we confirmed GR association, present in the 5’ UTRs of 7889 predicted mRNA targets, or in the entire sequences of 25,672 predicted mRNA targets (21% of the UniGene transcript pool) [81].

Overall, these data indicate that the GR can mediate GC action beyond its nuclear functions in transcriptional control, and that it may directly participate, via association with mRNA, in GC-mediated control of cytoplasmic mechanisms of gene expression. Though these data indicate an intriguing novel regulatory mechanism for the GR, much remains to be tested to understand its relevance in the global anti-inflammatory action of GC: for example, whether the functional role of the RNA motif is indeed to convey acceleration of mRNA decay, as documented for CCL2 and CCL7, and whether other GR motifs can be found in genes expressed upon inflammatory cell activation. To this end, multiple motifs have been documented for the RBP TIAR, which binds to U-rich motifs within the 3’UTR of its targets with high affinity when conveying translational repression, while its low-affinity binding to a C-rich element decreases upon cellular stress [83]. Likewise, it can be postulated that GC treatment, as well as a host of intra- and extracellular triggers, could modulate the GR binding to the RNA motifs in many ways. For example, this process could be modified by stimulus-induced changes in the RBP and microRNA potentially associated with GR in the RNP complex, and/or by action upon the association of GR-bound RNA with cytoplasmic foci of regulated mRNA decay and translation, such as P bodies and stress granules, where these functions are critically regulated during inflammation. On the structural level, much remains to be discovered on the cytoplasmic GR:mRNA association, starting from the identification of the GR domain interfacing with the GC-rich motif and the stoichiometry of this binding: what we know so far is that the GR lacks a conserved RNA-binding domain (RBD), present in other transcription factors that also bind to mRNA, like NF-90 and in nucleolin [84, 85]. In addition to a specific RBD, GR could also associate to other RBP components that bind directly to RNA.

Research on the role of miRNA in GC anti-inflammatory action is still in its infancy. The effect of GC in a mouse model of LPS-induced inflammation was minimal on global miRNA profiling in the lungs [86], as it was in a study of miRNA profiling in bronchial biopsies of mild asthmatics before and after therapy with inhaled glucocorticoids [87]. Given that miRNA expression is cell-specific, discrete changes may have been masked by the evaluation of tissue samples rather than purified cells. In line with this consideration, in fact, recent data indicate that GC potently inhibits in a macrophage cell line the expression of miR101, a suppressor of MKP-1 [88] and strongly repress miRNA expression during glucocorticoid-induced apoptosis of primary rat thymocytes [89].

## II. CONCLUSION

In conclusion, growing data indicate the existence of relevant, yet understudied molecular mechanisms involved in the physiologic control of the inflammatory response that could provide a different paradigm of anti-inflammatory approach, shifting from the control of master transcriptional mechanisms to that of posttranscriptional regulatory events. RNA-binding proteins and miRNA are increasingly seen as critical and targetable effector molecules of inflammation.

Considering that both molecular species coordinatley regulate functionally related pools of transcripts through common regulatory elements, intervening at the structural and functional interface between RBPs, miRNAs and their targets may convey a relatively specific anti-inflammatory outcome.
